# Gastric Outlet Obstruction Caused by PEG Tube Malposition: Contrasting Aspirates as a Diagnostic Clue

**DOI:** 10.1002/jgf2.70112

**Published:** 2026-03-16

**Authors:** Tatsuki Yamada, Takashi Inaba, Ilsu Jon

**Affiliations:** ^1^ Department of Internal Medicine Kasama City Hospital Kasama Ibaraki Japan; ^2^ Department of Family Medicine, General Practice and Community Health, Institute of Medicine University of Tsukuba Tsukuba Ibaraki Japan

A 56‐year‐old man with spinocerebellar degeneration, managed at home with a percutaneous endoscopic gastrostomy (PEG) tube for 2 years, developed sudden abdominal distension without fever. PEG feeding had been well tolerated until the day before. On abdominal examination, tympanic sounds were noted on percussion, and vomiting occurred during evaluation. For decompression, aspiration was attempted through the PEG tube; however, the aspirate differed in color from the vomitus, and vomiting persisted despite drainage through the PEG tube. Therefore, a nasogastric (NG) tube was inserted, which yielded fluid of the same color as the vomitus. Aspiration through the PEG tube yielded bile‐like yellow‐green fluid, whereas nasogastric (NG) tube aspiration produced dark brown gastric contents (Figure 1).

**FIGURE 1:**
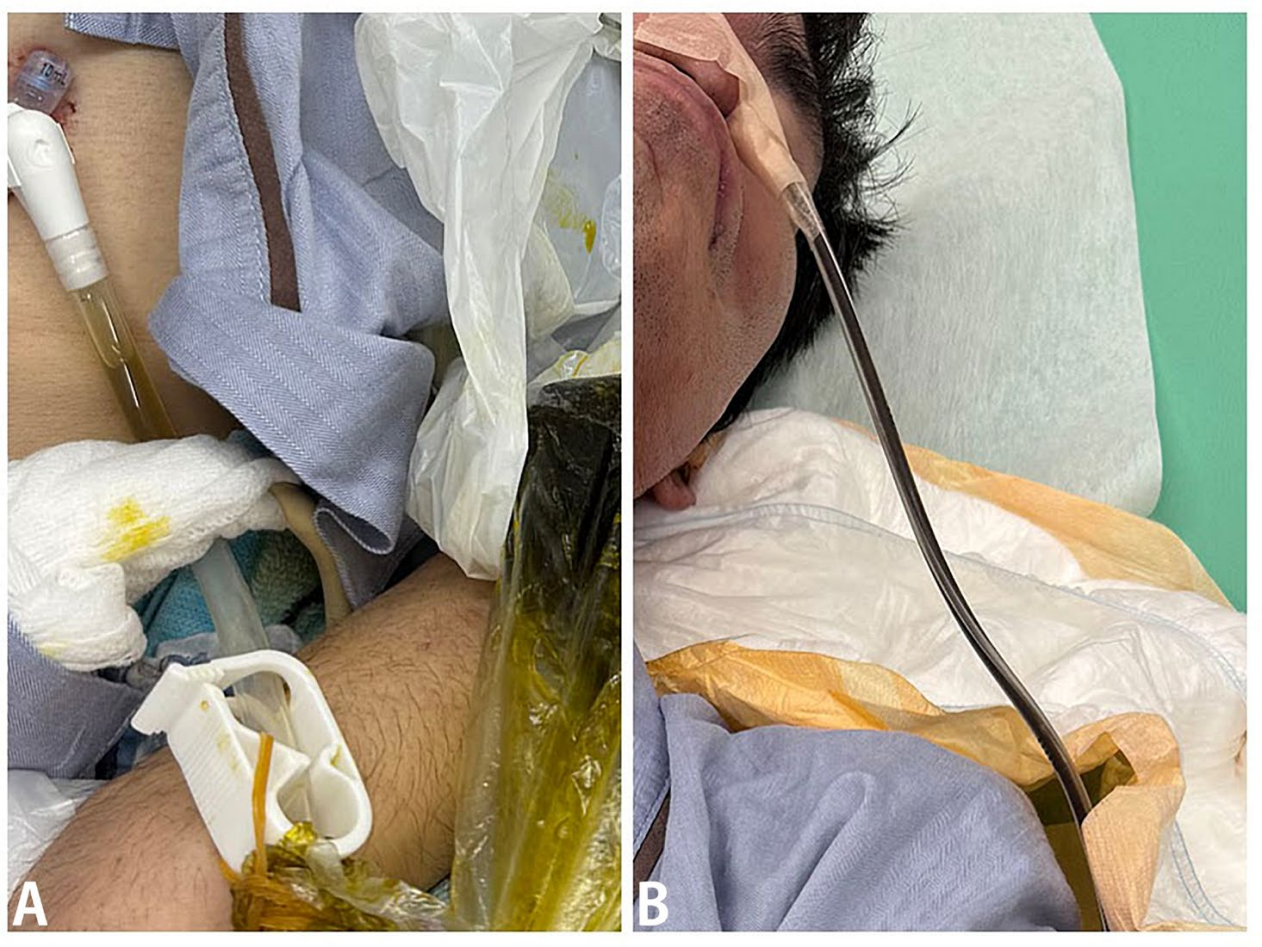
Contrasting aspirated fluids. (A) Bile‐like yellow‐green fluid from the PEG tube. (B) Dark brown gastric fluid from the nasogastric tube. The difference in color prompted suspicion of PEG tube malposition.

Abdominal computed tomography and endoscopy revealed that the PEG tube had migrated into the first portion of the duodenum (Figure 2). No signs of perforation or peritonitis were observed. Endoscopic repositioning of the PEG tube resulted in immediate resolution of symptoms.

**FIGURE 2:**
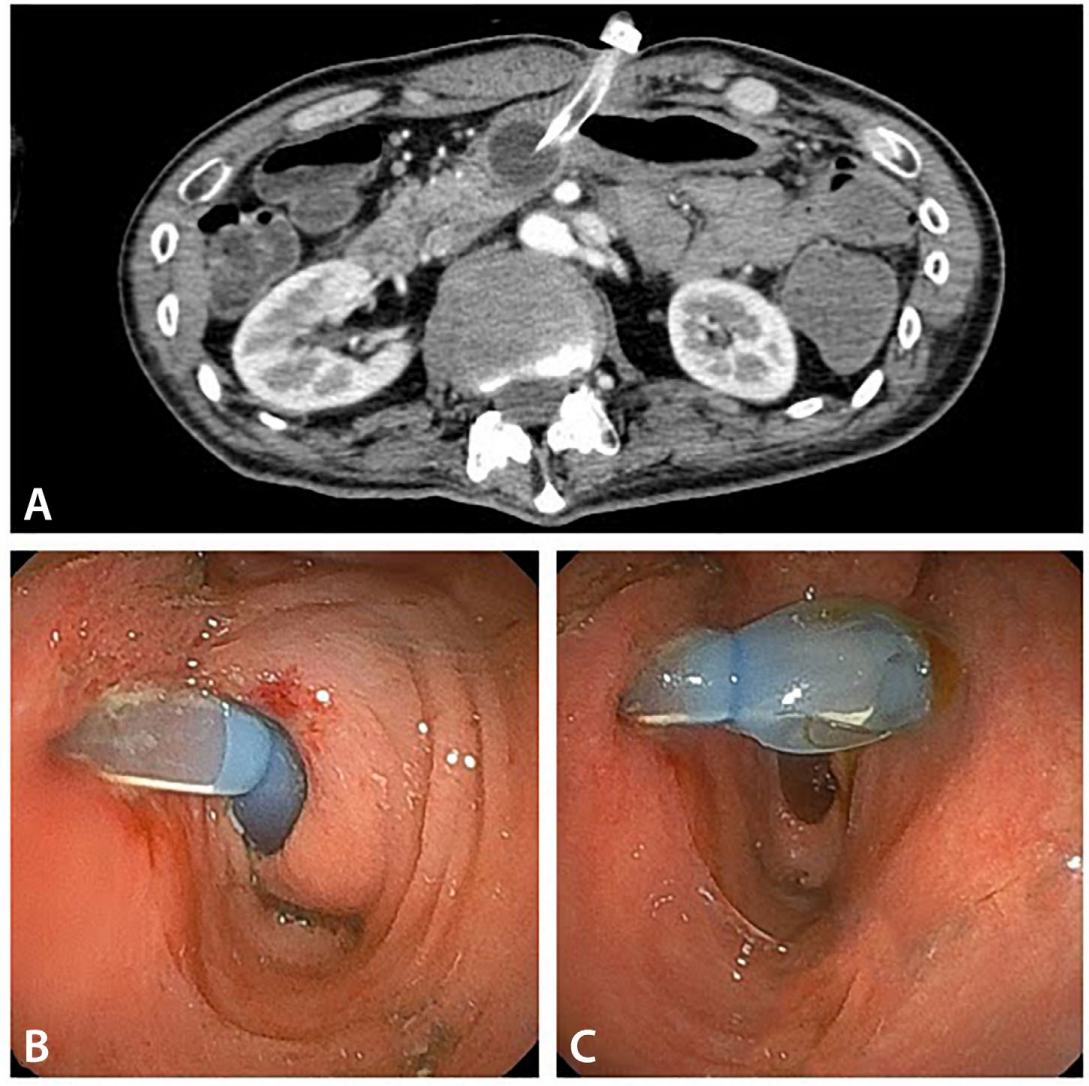
Imaging findings of PEG tube malposition. (A) Abdominal CT showing the PEG tube tip in the first portion of the duodenum. (B) Endoscopic image showing the PEG balloon tip malpositioned beyond the pylorus. (C) Endoscopic image showing the PEG balloon tip repositioned in the stomach.

Gastric outlet obstruction (GOO) is an uncommon complication of PEG tube placement, typically caused by tube migration into the pylorus or proximal duodenum [1, 2]. In this case, visual inspection of aspirate appearance provided an accessible bedside clue to the diagnosis. This simple observation is particularly valuable in home care settings, where advanced imaging may not be readily available.

## Author Contributions

**Tatsuki Yamada:** conceptualization; writing – original draft. **Takashi Inaba:** supervision; writing – review and editing. **Ilsu Jon:** writing – review and editing.

## Ethics Statement

The authors have nothing to report.

## Consent

Written informed consent was obtained from the patient to publish this article.

## Conflicts of Interest

The authors declare no conflicts of interest.

## Data Availability

Data sharing not applicable to this article as no datasets were generated or analysed during the current study.

## References

[1] S. P. Schrag , R. Sharma , N. P. Jaik , et al., “Complications Related to Percutaneous Endoscopic Gastrostomy (PEG) Tubes. A Comprehensive Clinical Review,” Journal of Gastrointestinal and Liver Diseases 16, no. 4 (2007): 407–418. 18193123

[2] J. Shah , T. Sunkara , K. S. Yarlagadda , P. Rawla , and V. Gaduputi , “Gastric Outlet and Duodenal Obstruction as a Complication of Migrated Gastrostomy Tube: Report of Two Cases and Literature Review,” Gastroenterology Research 11, no. 1 (2018): 71–74, 10.14740/gr954w. 29511412 PMC5827908

